# Interest in Quitting and Utilizing Quitline Services Among Long-Term E-Cigarette Users

**DOI:** 10.1080/10826084.2024.2447420

**Published:** 2025-01-09

**Authors:** Matthew Carrillo, Jessica Yingst, Gail Carmen D’Souza, Sitasnu Dahal, Sophia I. Allen, Jonathan Foulds

**Affiliations:** Department of Public Health Sciences, Penn State College of Medicine, Hershey, PA, USA

**Keywords:** Electronic cigarettes, quitline services, nicotine

## Abstract

**Background:**

Electronic cigarettes (e-cigs) contain fewer hazardous ingredients than traditional cigarettes, yet they still pose health hazards. This study evaluates experienced e-cig users’ quitting interest and Quitline utilization.

**Methods:**

In a 2012 (Wave 1) baseline survey, 1875 (28.9%) provided consent consented to future study contact. This study focused on a follow-up survey sent in 2022 (4). The main indicators assessed were participants’ were participants awareness, willingness, and motivations to utilize Quitlines to quit e-cigs. The Penn State Electronic Cigarette Nicotine Dependence Index (PSECDI) was used to assess the level of dependence on e-cigs. Descriptive statistics were used to examine the outcomes of interest. Additionally, a thematic analysis was performed to evaluate qualitative data.

**Results:**

Participants (*n* = 195) had a mean age of 52.4 (SD = 12.1) years, 64.6% (*n* = 126) were male, and the majority were Caucasian/White (88.2%, *n* = 172). About 42% (*n* = 82/195) of respondents had previously tried to quit e-cig use. Of these, more than half (63.4%, *n* = 52/82); had heard of Quitline; however, very few (9.8%, *n* = 8/82) were interested in utilizing Quitline services for assistance with quitting e-cigs. The themes that emerged included the impersonal nature of telephone counseling, lack of trust in external assistance, belief in participants’ ability to quit without help, and skepticism about the effectiveness of Quitline interventions.

**Conclusion:**

There was widespread reluctance to utilize Quitline cessation services among experienced e-cig users. To enhance engagement in cessation programs, such as Quitline, it may be helpful to consider specialized counseling and support tailored to the unique challenges among e-cig users.

## Introduction

Electronic cigarettes (e-cigs) heat a liquid containing nicotine into an aerosol that is inhaled by the user *via* a mouthpiece (Pesko et al., 2023). E-cigs allow the user to imitate the physical mechanisms of smoking a conventional cigarette including the hand-to-mouth motion and inhalation of vapor, with numerous refillable and rechargeable models widely available (Chen et al., 2023). Over the past decade, the use of e-cigs has increased substantially, especially among current smokers and recent quitters (Jones & Salzman, 2020). In 2014, almost 4% of US adults, including 15.9% of current and 22.0% of former smokers, reported daily or occasional use of e-cigarettes (Schoenborn & Gindi, 2015; King et al., 2013). In 2021, the prevalence of current e-cig users among adults aged 18 and older was 4.5%, with the highest usage observed among adults aged 18–24 at 11.0% (CDC, 2023; McNeill et al., 2022).

Studies have shown that e-cigs expose users to fewer harmful toxicants compared with traditional cigarettes (Shahab et al., 2017; Collier, 2017; Feeney et al., 2022), with one study showing that toxicants emitted from e-cig aerosol were 82% to 99% lower than from combustible tobacco cigarette smoke (Margham et al., 2016). Despite these findings, e-cigs are not harmless, and e-cig aerosols may contain several potentially harmful constituents such as tobacco alkaloids, aldehydes, volatile organic compounds, metals, and polycyclic aromatic hydrocarbons (Feeney et al., 2022; Ebersole et al., 2020), although for most of these, the concentrations are typically much lower than in cigarette smoke (Overbeek et al., 2020). Overall, there is limited evidence of the long-term health effects associated with e-cigs due to the short time that e-cigs have been used and the lack of long-term cohort studies available (Wasfi et al., 2022).

Given the ambiguous long-term health consequences linked to the use of e-cigs, research is needed to understand the interest in and ways to promote quitting among e-cig users. One study that examined e-cig cessation behavior among young adults found that 33.3% reported a quit attempt within the past year, 15.3% reported serious intentions to quit, and 54.2% reported general intentions to quit (Cuccia et al., 2021). Similarly, a study analyzing e-cig quitting intentions among individuals with and without a history of cigarette smoking revealed that 15.2% of participants reported a quit attempt within the past year and 60.7% reported future intentions to quit e-cigarettes (Palmer et al., 2021). Given that e-cigs are not harmless and there is interest in quitting, there is a need to develop and implement interventions to help individuals quit e-cigs (Amin et al., 2023).

Telephone Quitlines are most widely known for providing tobacco users with free evidence-based support and counseling to quit (Smith et al., 2021; Rosen & Steinberg, 2020). The North American Quitline Consortium (NAQC) 2022 survey of Quitlines found that 12,744 (5.4% of Quitline participants) individuals who exclusively use e-cigarettes received services from state Quitlines, while 46,808 (19.9% of Quitline participants) individuals who use dual-use e-cigarettes and other tobacco products received services from state Quitlines (NAQC, 2022). The Quitline providers services to both cigarette and e-cig user, and a secondary examination of US Quitline data showed that both groups of callers had similar success rates in quitting tobacco use, suggesting that established Quitline techniques for smoking cessation may also work for e-cig cessation (Short et al., 2023).

While tobacco cessation Quitlines are commonly used among cigarette smokers, and there is emerging evidence that they may help e-cig users, little is known about whether e-cigarette (e-cig) users are aware of Quitline services and whether e-cig users are interested in using them to discontinue e-cig use. Likewise, while there is substantial evidence on the effectiveness of Quitline for smoking cessation (Vickerman et al., 2021; Mok et al., 2023), the data on their success rates for helping individuals quit e-cigarettes is still limited. Only a handful of studies have directly evaluated the effectiveness of Quitline programs for e-cigarette users, with one pilot study indicating quit ranges to be from 20–30% (Vickerman et al., 2022). However, more research with larger samples over more extended periods is needed to establish the robustness of these findings. This study aims to evaluate interest in quitting e-cigs and using the Quitline among long-term ever e-cig users. Understanding long-term e-cig users’ willingness to utilize Quitline services will assist with informing effective cessation strategies and promoting public health initiatives in e-cig cessation.

## Methods

### Study sample

The participants in this mixed methods study were part of a longitudinal study of e-cig users. In 2012 (Wave 1), a link to the survey was posted on websites frequented by e-cig users including e-cigarette-forum.com and the NJOY website. At the end of the survey, individuals who wanted to participate in future studies could provide their contact information. Of the 6493 participants who answered the question regarding their readiness to participate in future research, 1875 (28.9%) provided consent to be contacted regarding future research, and 28.7% (*n* = 1863) supplied their email and/or phone numbers allowing for future contact.

Follow-up surveys were sent in January 2017 (Wave 2), June 2019 (Wave 3), and June 2022 (Wave 4). Email invitations were sent to the 1863 participants who agreed to future contact. Weekly email reminders were sent up to four times, along with telephone call reminders for those who did not respond. Of the 1,863 who consented to be re-contacted, 649 (34.8%) participated in the wave 2 survey, 573 (30.8%) participated in the wave 3 survey, and 274 (14.7%) participated in the wave 4 survey. This study focused on data collected at Wave 4 in 2022, with our small convenience sample only consisting of participants who responded to questions related to quitting e-cigs 195 (10.5%).

### Measures

Our primary measures evaluated participant awareness of Quitlines for e-cig cessation, perceptions of Quitline services, consideration of using Quitlines to quit e-cigs, and reasons for Quitline utilization. Participants were also asked about basic demographics and their preferred e-cig device and its characteristics. The Penn State Electronic Cigarette Nicotine Dependence Index (PSECDI) was used as a measure of e-cig dependence. This questionnaire was developed to measure e-cig dependence and generates a dependence score between 0 and 20 (0–3 =not dependent, 4–8 low dependence, 9–12 medium dependence, 13+ = high dependence) (Foulds et al., 2015).

Participants were asked “Have you used an electronic cigarette in the past 30 days?” (yes/no) and “Have you tried to quit using your e-cig?” (yes/no). Those who reported a previous quit attempt were asked the following, “Have you ever heard of telephone Quitlines for help with quitting tobacco use?” (yes/no) and “Would you ever consider calling the Quitline for help quitting e-cig use?” (yes/no). The complete list of structured questions used to gather qualitative responses is represented in Table 1.

### Data analysis

The survey data were collected and managed using REDCap electronic data capture tools hosted at the Penn State Clinical & Translational Research Institute, Pennsylvania State University. Statistical analyses were performed utilizing SAS version 9.4. Our sample included e-cig users who answered the question about attempts to cease using e-cigs (*n* = 195), meaning that the sample included both successful and unsuccessful attempters. Descriptive statistics were utilized to describe the sample and characteristics of e-cigarette devices and use patterns of the current users. In addition, the study also examined rates of successful cessation using e-cigarettes and the level of awareness and utilization of Quitlines among individuals (Table 2).

Researchers M.C and J.Y. utilized MAXQDA, a specialized software designed to examine qualitative data, to conduct a deductive qualitative thematic analysis. We utilized a deductive approach as we initiated the data procedure by using preexisting themes based on our previous knowledge. We used an iterative process to refine the preexisting codes during the coding process, including the alteration and division of different codes as required. After using an iterative process to develop and refine codes, researchers M.C. and J.Y. performed a comprehensive examination of the encoded data to find dominant themes and patterns. After establishing our primary themes, M.C. and J.Y. continued to develop sub-themes that provided additional clarity on the relationships and interactions within the central topic Subsequently, we developed narratives, which analyzed each theme, revealing its inherent significance and supporting assertions with evidence from the facts (Morse, 2015).

## Results

Current e-cig users (*n* = 136, exclusive and dual users) reported using their e-cig a mean of 18.5 times per day (SD = 17.7), with a mean PSECDI score of 8.6 (SD = 4.0), indicating overall low to medium dependence. Most current users reported use of a mod device (64.7%, *n* = 88) or a pod-mod device (18.4%, *n* = 25). Among those who reported the nicotine concentration of their liquid (*n* = 118), the mean was 15.1 mg/ml (SD = 16.2) (range 0–60 mg/ml). The most common flavors used in the past 30 days were fruit (45.6%, *n* = 62), tobacco (29.4%, *n* = 40), candy/chocolate/other sweets (26.5%, *n* = 36) and menthol or mint (24.3%, *n* = 33). Among those who had quit e-cigs (*n* = 59), the overall mean time since last e-cig use was 1,324 days or 43.5 months (SD = 1028.7).

More than a third of participants (42.1%, *n* = 82) had ever tried to quit or quit using their e-cig. Among those who have ever tried to quit or quit (*n* = 82), more than half (63.4%, *n* = 52/82) had heard of the Quitline but of these, only 9.8% (*n* = 8/82) reported ever considering calling the Quitline to help quit their e-cig use.

The qualitative survey responses were categorized into prevalent themes related to why e-cig users did not consider using the Quitline, including: 1. Perceived impersonal nature of telephone counseling; 2. Lack of trust; 3. Confidence in self-ability to quit; 4. Preference for utilizing other resources; and 5. E-cigs are not perceived to be addictive. The number of subthemes within each theme varied based on the responses from participants, and each subtheme had 1 to 2 participant quotes corresponding to it. Supplement Table 1 shows the themes found within our thematic analysis, subthemes developed within the main themes, and examples of responses corresponding to those topics. It is important to note that these qualitative findings are based on a limited subset of participants and should not be generalized to the entire sample. Instead, they provide insights into the perspectives of this specific group. Furthermore, the quotes shown in Supplemental Table 1 are not an exhaustive list but rather highlight some of the most relevant and salient responses based on our identified themes and subthemes.

### Theme 1: Perceived impersonal nature of telephone counseling

The central theme that arose was the perceived ineffectiveness associated with telephone counseling. For example, numerous participants conveyed their unease about engaging in conversations over the phone with strangers. One participant said, *“Talking on the phone to a stranger about quitting seems weird.” [35 years old, Caucasian, Male]*. Many participants also acknowledged experiencing social discomfort and unease when discussing their issues with unfamiliar individuals. One participant remarked, *“I’m socially awkward, and talking to random people about my problems is not something that I’m cool with.” [22 years old, Caucasian, Male].*

A significant number of participants also conveyed a preference for encounters that were more individualized and consistent. One participant highlighted the importance of having a dedicated individual to contact, saying, *“Unless there was a dedicated person I would be able to call and follow up with rather than calling a different person each time.” [21 years old, Middle Eastern, Male].*

Certain participants expressed skepticism over the efficacy of Quitline personnel, presuming that they may consist of volunteers who adhere to predetermined prepared responses. One participant asserted,

“Talking on the telephone to, what is most likely a volunteer with a provided script of what to say, is not going to help me in any way to quit smoking e-cigs.” [44 years old, Caucasian, Female]

### Theme 2: Lack of trust

Another prominent theme was distrust of external help. Participants articulated apprehensions over the perception of being under surveillance, subject to manipulation, or having their usage patterns and challenges recorded in publicly accessible archives. One participant said, *“I don’t want to feel like people are checking in on me about what I do with my body. I don’t want to feel manipulated. I don’t want public records of my use or struggle.” [24 years old, Caucasian, Male].*

### Theme 3: Confidence in self-ability to quit

Self-confidence in quitting arose as another common theme as numerous individuals conveyed a notable inclination toward independence and self-sufficiency, as one individual stated, *“I don’t tend to look to others for help with anything in life…” [33 years old, Caucasian, Male].* Certain participants outright rejected the necessity of Quitline’s services *“I solve my own problems and prefer not to rely on others…” [27 years old, Caucasian, Male].* Many participants believed that the act of quitting could not be imposed onto individuals and that individuals would only embark on the process of stopping when they experienced a sense of internal readiness. As one participant stated, “You won’t quit until you are ready to quit.” [23 years old, Caucasian, Male]

### Theme 4: Preference for internet resources

Several individuals indicated a preference for using online tools to obtain knowledge and seek support, *“The internet is a better resource…” [27 years old, Caucasian, Male]”.* They also emphasized the presence of digital resources that can be utilized to investigate and obtain relevant information about the cessation of e-cigarette usage. For example, one participant reported, *“The internet is available for a search of available resources.” [29 years old, Caucasian, Female].*

### Theme 5: E-cigs not perceived to be addictive

Notably, a subset of subjects exhibited a lack of perception of the addictive nature of e-cigarettes. The participants referred to infrequent utilization of e-cigarettes, primarily for relaxation, which was distinguished from chronic or addictive smoking patterns. One participant stated,

“I feel like e-cigs are much easier to quit than normal cigarettes. I know, I smoked for 20 years and then used e-cigs over 8 years to quit completely. I am living proof of exactly how it is supposed to work.” [32 years old, Caucasian, Male].

## Discussion

This study investigated long-term e-cig users’ interests in quitting and using the Quitline services for assistance. Our small convenience sample was representative of the initial sample of participants interested in future research studies, primarily within demographics. Our results from our small convenience sample indicated that more than half of e-cig users who had ever tried to quit had heard of the Quitline; however, very few e-cig users were interested in utilizing Quitline services for assistance with quitting e-cigs. Similarly, a population-based study revealed that e-cig users have a high level of awareness for Quitline services, but only a small proportion of them had attempted to quit smoking by using Quitline’s (Kinnunen et al., 2015).

Many participants in our study believed that using the Quitline was an ineffective approach because they felt the interactions over the phone with multiple providers was impersonal. Unsurprisingly, participants desired individualized support tailored to their specific circumstances and requirements. Specifically, they preferred one-on-one interactions with one dedicated Quitline personnel instead of multiple providers, suggesting that individual attention would provide more practical guidance and comprehension. Participants perceived Quitlines to lack a sense of personalization, which is also commonly shared by cigarette users considering the Quitline. For example, one qualitative study on Quitline perceptions among smokers also found that users reported the interactions as generic and not tailored to the caller (Waters et al., 2015). Another study discovered that tailored behavioral support was one of the most successful cessation techniques.

Another prominent theme was participant’s strong belief in their self-efficacy to quit e-cig use. Many participants stated that if they wanted to quit, they could, linking to participant beliefs that e-cigs are not addictive. These beliefs decreased their desire to seek assistance from the Quitline or any other external source. These findings suggest that despite the majority of participants familiarity with Quitline services, most e-cig users prefer to quit on their own, or self-reliance perceptions may be preferred over Quitline cessation services. In addition, respondents expressed a preference for Internet resources. This could be due to a higher level of familiarity and convenience in accessing such resources by preferring to pursue information and assistance through online platforms. The availability of a vast selection of online materials and support communities may be more appealing to their preferences and give them a sense of autonomy and control during their voyage to cease smoking.

### Increasing Quitline use among E-cig users

Several strategies can be implemented to increase the desirability of Quitline services for e-cig users. For example, raising awareness of Quitline’s availability as a resource for e-cigarette cessation is crucial (Vickerman et al., 2021). This can be accomplished through targeted promotional campaigns, advertisements, and outreach initiatives emphasizing Quitline’s availability as a resource for e-cig cessation. Although most participants indicated that they were aware of the Quitline services, there are still many users not aware of the free service. In addition, since interest in quitting is transient, frequent reminders of the availability are important. For example, findings from a randomized clinical trial showed that a personalized and interactive text message intervention effectively facilitated the cessation of vaping (Graham et al., 2021). In addition, using a variety of communication channels, such as social media platforms, vaping-related websites, and community forums, can assist in reaching a larger audience of e-cigarette users (Kaufman et al., 2010).

The effectiveness of the Quitline was viewed with skepticism by the majority of participants, who questioned the ability of its staff to assist them in ceasing vaping; therefore,

Quitline services can further enhance their offerings by providing e-cig users with specialized counseling and support tailored to their unique challenges. This may entail educating Quitline counselors on e-cigs, their components, and usage patterns (Cummins et al., 2016; McMillen., 2015). Given the rapidly changing landscape of e-cigs and the prevalence of misinformation, it is essential to keep Quitline clinicians well-informed about the newest research on e-cigarettes. By providing e-cig users with knowledgeable and empathetic counselors who can address their specific concerns and barriers, Quitline’s can become more alluring and pertinent. In order to make Quitlines more appealing, it is crucial to prioritize participants’ inclination for personalized engagement, adapting support to their unique requirements, and ensuring confidentiality.

Future research should investigate alternate methodologies for gathering longitudinal data on this group to expand upon the findings of this study. Constructing studies that include numerous data-collecting time points, such as biannual or quarterly follow-ups, is advisable to monitor e-cigarette users’ quitting behaviors over an extended duration (Zhuang et al., 2016). Furthermore, integrating self-report surveys with objective metrics, such as biomarkers or digital monitoring of e-cigarette consumption, may yield more complete data and reduce memory bias (Lorkiewicz et al., 2019).

To address the underutilization of Quitline services, developing cessation interventions that blend self-guided digital tools with intermittent access to human counseling may be a promising approach for those who value autonomy but could benefit from occasional expert support. Future research might investigate the efficacy of app-based cessation programs that provide real-time feedback, customized material, and behavioral support, as these interventions are more appealing to e-cigarette users who choose technology-driven solutions than conventional Quitline services (Lyu et al., 2022). Enhancing studies to comprehend the obstacles and enablers of Quitline participation across various subpopulations of e-cigarette users would help guide the creation of more inclusive and effective cessation resources (Colston et al., 2022).

Overall, the development of multi-channel cessation tools, encompassing digital, phone-based, and in-person assistance, may address deficiencies in existing Quitline programs and enhance quitting results for e-cigarette users (Lyu et al., 2022). Future studies must evaluate the efficacy of these innovative cessation strategies compared to conventional Quitline programs. Furthermore, examining the integration of these technologies with the current Quitline infrastructure to establish a holistic, multi-modal strategy for e-cigarette quitting support is essential.

### Strengths and limitations

A strength of this study is that our sample consisted of a cohort of e-cig users followed over many years, a group for which data is currently limited (Sobieski et al., 2022). In addition, few studies have focused on assessing participants’ perspectives on using Quitline services to quit e-cigs (Vickerman et al., 2021). Despite the study’s strengths, there are several limitations, including a small convenience sample and reliance on self-reported data, which introduces the potential for recall and social desirability biases. However, this study may be used as a model for developing larger studies of individuals who use e-cigs and may consider using Quitlines for support. Another limitation pertains to the methodology employed for data collection. Specifically, we did not conduct interviews; therefore, future studies should prioritize the implementation of semi-structured qualitative interviews to obtain comprehensive insights into their e-cig use.

## Conclusion

This study assessed awareness of tobacco cessation Quitlines, perceptions of Quitline services, and overall Quitline utilization among long-term e-cig users. Within our small convenience sample, most participants had no desire to contact Quitlines for assistance with quitting e-cigs despite high awareness of these cessation services. Common themes that emerged throughout participants’ responses for having little interest in using Quitlines were distrust of outside assistance, self-efficacy in quitting e-cig use, perceptions that e-cigs are not addictive, preference for individualized care, and preference for internet resources. It may be helpful to inform e-cig users that most modern Quitline services provide callers with the option of using texting-based or online assistance in addition to (or in place of) telephone counseling and there is growing evidence that these interventions can increase callers’ chances of successfully quitting (Graham et al, 2021)

## Supplementary Material

Supplemental Materials

Supplemental data for this article can be accessed online at https://doi.org/10.1080/10826084.2024.2447420.

## Figures and Tables

**Table 1.** Survey questions.

**E-Cig Use & Quitline Questions** 1. Have you used an electronic cigarette in the past 30 days? 2. Have you ever tried to quit using your e-cig? 3. Have you ever heard of telephone Quitlines for help with quitting tobacco use? 4. Would you ever consider calling the Quitline for help quitting e-cig use? 5. Why or why not? Please explain your answer.
**Preferred E-Cig Device Use & Characteristic Questions** 1. From the above options, which type of device do you use the most/is your most preferred device? 2. Which of the following flavors have you used in the past 30 days? 3. What is the mg/ml nicotine level?
**Demographic Questions** 1. What is your current age? 2. Are you a man or a woman? 3. Do you consider yourself to be Hispanic/Latino? 4. What race or ethnicity best describes you?

**Table 2.** Summary of participant demographics.

characteristics	*n* (%) or mean ± SD

**Age**	52.4 ± 12.1
**Sex**	
Male	126 (64.6%)
Female	66 (33.9%)
Missing	3 (1.54%)
**Race**	
Caucasian/White	172 (88.2%)
Other	20 (10.3%)
Missing	3 (1.5%)
**Ethnicity**	
Hispanic/Latino	6 (3.1%)
Not Hispanic/Latino	179 (91.8%)
Chose not to answer	2 (1%)
Missing	8 (4.1%)
**Current Tobacco Use Status**	
Current user	136 (69.7%)
Prior user	59 (30.3%)
