# Supplementary material for: Interest in Quitting and Utilizing Quitline Services Among Long-Term E-Cigarette Users
Source: Subst Use Misuse. Author manuscript; Available in PMC 2025 Sep 4. (PMC12410069; doi:10.1080/10826084.2024.2447420)
Supplement: Supplemental Materials [file NIHMS2104335-supplement-Supplemental_Materials.docx]

**Supplemental Table 1: Themes and sub-themes of participant’s quotes**

| **Theme 1: Perceived ineffectiveness of telephone counseling** | |
| --- | --- |
| **Subtheme:** Don’t like talking over the phone | “Talking on the phone to a stranger about quitting seems weird.” [35 years old, Caucasian, Male]  “Not effective for me and I don’t like talking on the phone.” [40 years old, Caucasian, Male] |
| **Subtheme:** Social Anxiety | “I'm socially awkward and talking to random people about my problems is not something that I'm cool with.” [22 years old, Caucasian, Male] |
| **Subtheme:** Preference for individual interaction | “Unless there was a dedicated person I would be able to call and follow up with rather than calling a different person each time.” [21 years old, Middle Eastern, Male] |
| **Subtheme:** Doubt among Quitline personnel | “Talking on the telephone to, what is most likely a volunteer with a provided script of what to say, is not going to help me in any way to quit smoking e-cigs.” [44 years old, Caucasian, Female] |
| **Theme 2: Lack of Trust** | |
| **Subtheme:** Distrust of external help | “I don't want to feel like people are checking in on me about what I do with my body. I don't want to feel manipulated. I don't want public records of my use or struggle”. [24 years old, Caucasian, Male] |
| **Theme 3: Confidence in self-ability to quit** | |
| **Subtheme:** Independent | “I don't tend to look to others for help with anything in life.” [33 years old, Caucasian, Male]  “I solve my own problems and prefer not to rely on others.” [27 years old, Caucasian, Male] |
| **Subtheme:** Self-readiness as a prerequisite for quitting | “You wont quit until you are ready to quit.” [23 years old, Caucasian, Male]  “I've tried. It's pretty silly. You are either ready to quit or you aren't. No one else can make that decision for you.”[29 years old, Caucasian, Male] |
| **Theme 4: Preference for utilizing other resources** | |
| **Subtheme:** Internet-based resources | “The internet is available for a search of available resources.” [29 years old, Caucasian, Female]  “The internet is a better resource.” [27 years old, Caucasian, Male] |
| **Theme 5: E-cigs not perceived to be addictive** | |
| **Subtheme:** E-cigs perceived to be easier to quit then cigarettes | “I feel like e-cigs are much easier to quit than normal cigarettes. I know, I smoked for 20 years, and then used e-cigs over 8 years to quit completely. I am living proof of exactly how it is supposed to work.” [32 years old, Caucasian, Male] |
